# Supplementary material for: Quantitative modeling of regular retinal microglia distribution
Source: Sci Rep. 2021 Nov 22;11:22671. doi: 10.1038/s41598-021-01820-3 (PMC8608893; doi:10.1038/s41598-021-01820-3)
Supplement: Supplementary file 1 — Supplementary Information. [file 41598_2021_1820_MOESM1_ESM.pdf]

Supporting information

Model of microglia process length distribution

Based on the experimentally observed motion of the process, we modeled the dynamics of process length distribution $u(x,t)$ using advection and degradation terms as follows:

$$\frac{\partial}{\partial t}u(x,t) = -g\frac{\partial}{\partial x}u(x,t) - p\,u(x,t)\ . \tag{5}$$

g is the velocity of process extension and p is the collapse probability of process. By the model (5), the steady-state of length distribution $u(x,\infty)$ can be described as follows:

$$u(x,\infty) = U_0\,e^{-\frac{p}{g}x}\ . \tag{6}$$

U_0 represents a certain constant. $u(0,\infty)$ is exponential distribution, indicating there is a linear relationship between the log of process number and process length.

The sum of the process length is described by the definite integral as follows:

$$\int_0^\infty xu(x,\infty)dx = \int_0^\infty xU_0e^{-\frac{p}{g}x}dx = U_0\frac{g^2}{p^2}\ . \tag{7}$$

U_0 are obtained by linear fitting equation ($U_0 = 7.6$ in avascular area and $U_0 = 7.7$ in vascular areas). $U_0\frac{g^2}{p^2}$ are 1.0×10^5 µm vs. 1.1×10^5 µm, and the sum of the process length from direct measurements are 1.1×10^5 µm vs. 1.3×10^5 µm, respectively. This similarity shows that the model captures the characteristics of the actual process distribution.

Obtaining simulation parameters from previous literatures

- c: We calculated the ATP chemotaxis coefficient using the published data of ATP chemotaxis of microglia in the Dunn chamber[8].

- h: We determined the ATP decay coefficient by fitting the data that ATP decay of homogenized retina[32]. The fitting equation was as follows:

$$a(t) = c_0 + c_1t + c_2e^{-ht}\ (c_n : \text{constant}). \tag{8}$$

- p: The basal extracellular ATP production rate[Sup1] is calculated by steady state concentration of ATP (a_0) and ATP decay rate h. We used extracellular ATP of the bovine retina (6.2 ± 0.7 nM[Sup2]) as a_0. We obtain p by steady state equilibrium $0 = p - ha_0$. Uptake by microglia is small and neglected in this calculation.

- σ: Davis *et al.* reported the exclusion radii (58.6 ± 1.4 µm) and the average NND (40 ± 1.0 µm) in adult mouse retina[17]. We measured the average NND (31 ± 0.12 µm, n = 7053, Fig. 3) in P5 mouse retina; thus, we assumed microglia repulsion radius is 45 µm, considering the ratio of their report[17].

- a_w: Intracellular ATP is millimolar order[33]. We set production of ATP at the wound site so that a_w becomes 1/300 of intracellular ATP.

Screening repulsion-related genes from single-cell RNASeq data.

It is known that there is a regional difference in microglia cell density. Microglial cell density is lowest in the cerebellum and highest in the hippocampus[57]. This may be correlated to the mutual repulsion of microglial cells.

At first, we obtained differentially expressed genes of microglia from different regions of CNS (GSE123025)[60]. DEGs between the cerebellum and other regions of CNS were provided in supplemental files[60] ($FDR < 0.05$).

- Genes upregulated in cerebellum: Jun, Junb, Dusp1, Fos, Klf2, Egr1, Bcl2a1d

- Genes downregulated in cerebellum: C230081A13Rik, Ddx27, Kctd21, Pecam1, Cog5, Hist1h1e, Ddit4, Fcrls

Gene enrichment analysis using these DEGs with G: profiler[Sup3] did not show cell movement-related pathways. We also utilized clustering data of the original publication (provided by Dr. Tristan Qingyun Li, Washington University) to obtain DEGs between the cerebellum and hippocampus (Mann-Whitney test, Bonferroni correction, $p < 0.05$).

- Upregulated genes: Egr1, Fos, Jun, Klf2

- Downregulated genes: Arglu1, Cst3, ERCC-00074, Fam102b, Fcrls, Marcks, Tmsb4x, Tyrobp

Again, gene enrichment analysis using these DEGs did not show cell movement-related pathways. However, among these DEGs, Egr1 and Klf2 were detected in both cases. They are relatively well studied and are not general housekeeping genes. This information may facilitate further candidate-approach studies.

References for Supporting Information

[Sup1] R. Corriden and P. A. Insel. Basal Release of ATP: An Autocrine-Paracrine Mechanism for Cell Regulation. *Science Signaling*, 3(104):re1–re1, jan 2010.

[Sup2] Claire H. Mitchell David Reigada, Wennan Lu, May Zhang, D. Reigada, W. Lu, M. Zhang, and C.H. Mitchell. Elevated pressure triggers a physiological release of ATP from the retina: Possible role for pannexin hemichannels. *Neuroscience*, 19(2):396–404, nov 2008.

[Sup3] Uku Raudvere, Liis Kolberg, Ivan Kuzmin, Tambet Arak, Priit Adler, Hedi Peterson, and Jaak Vilo. G:Profiler: A web server for functional enrichment analysis and conversions of gene lists (2019 update). *Nucleic Acids Research*, 47(W1):W191–W198, 2019.
